# Some Phases of Dental Literature

**Published:** 1889-08

**Authors:** W. Storer How

**Affiliations:** Philadelphia, Pa.


### SOME PHASES OF DENTAL LITERATURE.[1]

BY W. STORER HOW, D.D.S., PHILADELPHIA, PA.

The practice of dentistry as a distinct department of medico-surgical art is of such comparatively modern origin that its literature is relatively recent; but its history in its entirety has yet to be written. A very creditable though hastily compiled account of the rise and progress of American dentistry, including a glance at its ancient origin, was embodied in a small volume by Dr. James E. Dexter, under the auspices of the American Academy of Dental Science, in 1876; but a complete condensed record of the development of dentistry from the beginning is still wanting, and is a great desideratum. There are in this country libraries containing in large measure the materials for such a work, and it would seem that the requisite professional ability, scholarship, and industry should not be lacking for the undertaking. Indeed, it is the chief object of this brief paper to arouse a renewed interest in the subject, and lend, it may be, some incitement to the accomplishment of the object which is so obviously a pressing need of the present day. This need becomes the more apparent when we consider the already established position of dentistry in the ranks of the recognized professions, in which technical literature forms so prominent a part.

The subdivision of professions into specialties, wherein attention is focussed upon the different branches or departments of the callings, with a correspondingly increased minuteness of observation and differentiation, leads, furthermore, to an expansion and elaboration of descriptive terms which render it well-nigh impracticable for any single professor to become familiar with the minutiæ of the whole field.

---

[1] Read before the New Jersey State Dental Society, Asbury Park, July 19, 1889.

For instance, the medical practitioner of fifty years ago was compelled to combine in himself the functions of the physician, surgeon, gynæcologist, dentist, oculist, aurist, dermatologist, druggist, chemist, etc., as indeed must he of to-day be in sparsely-populated sections; but let such an one enter the library at Washington and look over the classified contributions to these branches in their direct relationships to the art of human healing, and he will stand in astonishment before the unfamiliar terms of the proficients who have independently and assiduously cultivated every part of the field represented by this mass of medical literature.

Dentistry is in nowise behind the other groups of the advancing medical membership in practical progress; yet there is reason to think it desirable that greater efforts should be made to attain a higher degree of cultivation in the art of permanently recording the degrees of progress that constitute the real value of the literature which is to become history for the professional men of the succeeding ages. It appears quite probable that the prevalent eagerness of everybody to get ahead of everybody else, with everything, is overmastering the old-time conservative injunction to make haste slowly, and there is therefore no time reserved for a record of the ways and means by which the advance may have been made. On the other hand, it would sometimes seem that the infectious methods of the newpaper reporters have been caught by professional pioneers who, by return mail or telegraph, put in print their supposed or wished-for discoveries, lest others should anticipate them and their own news be deemed stale. Alas! it too often turns out that the things realized or guessed at so hastily were already a part of the literature of the past, but had been lost to sight because of their burial in some ephemeral paper or journal, or even in a persistent journal, whose bound volumes have no classified general index by which the contents of the series might be topically reviewed by the thoughtfully studious practitioner. Moreover, such bound volumes are possessed by comparatively few, and even these, if we judge by the absence in current literature of anything like topical research, are both forgetful and neglectful of the stores of interesting and practical information at their command.

The possession or use of a library which embraces every obtainable book or journal relating to his specialty is one of the unmistakable signs of a studious professional man, and is also one of the indispensable accompaniments of a truly professional career. The fact that some modern dental books, of large size and high cost, have an extensive sale is indicative of a growing taste for professional information directly applicable to daily practice.

For example, "The American System of Dentistry," in its three large volumes, is comprehensive yet specific in exposition of modern theories, discoveries, and modes of practice; but the intelligent practitioner desires knowledge of all the superseded or discarded means and processes of dental practice, in order that he may not waste time in repeating abandoned methods, and also in order that in emergencies he may avail himself of some old expedients that had been proven to be of special use, though not adapted for general employment. A library is therefore a necessity as well as a sign of a thoroughly-equipped professional specialist, such as the dentist ought to be. As a fact, he is not yet a book-buyer, a journal-reader, and a preserver. Comparatively few have even the standard text-books, and fewer yet have bound copies of established American journals, to say nothing of foreign books and periodicals published in the English and other languages.

Such discreditable dearth of dental literature among would-be professional men is by no means an encouraging feature of the prospects for the future of dentistry as a specialty in the art of healing. Nevertheless, it is entirely practicable to engage in concerted and persistent endeavors to develop and foster among dentists a due appreciation of the professional necessity of cultivating an intimate acquaintance with past and present dental literature. As one means to this end, the formation and maintenance of society libraries is worthy of immediate and systematic consideration. Another—the history already referred to—may be expedited by society action; indeed, the present is a good time and this Society a good medium for initiating action with this object in view.

The editor of the "American System of Dentistry" has therein shown competency for the desired work, and probably has such relations with well-known publishers of professional books that unusual facilities for forwarding the undertaking would be at his command.

So far as the writer knows nothing has been said or intimated on this subject to Dr. Litch; but the suggestion of his name is made with the design of inducing prompt and definite action in a matter of such obvious importance to the profession. Spasmodic efforts in this direction have been made in somewhat recent years, but practically nothing has been accomplished.

Indeed, nothing of this kind can be accomplished unless there shall be aroused a vivid perception of the fact that the honor of the dental profession is jeopardized by further delay in putting into a generally accessible printed form the main facts in the development of cosmic dentistry from its beginning to the present time.

Some very valuable preliminary work has been done by Mr. George Crowley in his "Dental Bibliography" of 1885, which is a standard reference list of books on dentistry, published throughout the world, from 1536 to 1885. These are arranged chronologically, serially numbered, and supplemented with a complete cross-reference to authors. The work is thus very conveniently adapted for use by the historian as well as by the studious dentist who has a library.

The monthly bibliography of dental literature, compiled by J. Melvin Lamb, M.D., and published in the *Dental Cosmos*, is another aid to the historian that cannot fail to be duly appreciated by him.

The work should of course be printed in English as most nearly approximating a universal language, but if condensed into a few volumes, of not too large size, there will no doubt follow translations into transatlantic tongues, as notably the French and German.

With this mere epitome of what might readily be said on the subjects introduced, the matter, which may well be deemed one of great consequence to dentistry as a profession, is laid before this prominently progressive and assiduous Society in the confident expectation that proper, immediate, and effective action will be taken.

